# miR‐15b‐5p facilitates the tumorigenicity by targeting RECK and predicts tumour recurrence in prostate cancer

**DOI:** 10.1111/jcmm.13469

**Published:** 2018-01-24

**Authors:** Ran Chen, Lu Sheng, Hao‐Jie Zhang, Ming Ji, Wei‐Qing Qian

**Affiliations:** ^1^ Department of Urology Huadong Hospital Affiliated to Fudan University Shanghai China; ^2^ Shanghai Dingdian Biotechnology Limited Company Shanghai China

**Keywords:** miR‐15b‐5p, growth, invasion, prostate cancer, RECK

## Abstract

MicroRNAs (miRNAs) have been reported to participate in many biological behaviours of multiple malignancies. Recent studies have shown that miR‐15b‐5p (miR‐15b) exhibits dual roles by accelerating or blocking tumour progression. However, the molecular mechanisms by which miR‐15b contributes to prostate cancer (PCa) are still elusive. Here, miR‐15b expression was found significantly up‐regulated in PCa in comparison with the normal samples and was positively correlated with age and Gleason score in patients with PCa. Notably, PCa patients with miR‐15b high expression displayed a higher recurrence rate than those with miR‐15b low expression (*P* = 0.0058). Knockdown of miR‐15b suppressed cell growth and invasiveness in 22RV1 and PC3 cells, while overexpression of miR‐15b reversed these effects. Then, we validated that RECK acted as a direct target of miR‐15b by dual‐luciferase assay and revealed the negative correlation of RECK with miR‐15b expression in PCa tissues. Ectopic expression of RECK reduced cell proliferation and invasive potential and partially abrogated the tumour‐promoting effects caused by miR‐15b overexpression. Additionally, miR‐15b knockdown inhibited tumour growth activity in a mouse PCa xenograft model. Taken together, our findings indicate that miR‐15b promotes the progression of PCa cells by targeting RECK and represents a potential marker for patients with PCa.

## Introduction

PCa is one of the most commonly diagnosed malignant tumours in men and is responsible for the high‐cancer death 1. Although many treatment strategies such as surgery, hormonal therapy, radiation therapy, chemotherapy and targeted therapy have been applied for patients with PCa, most of them present with tumour metastases at the time of diagnosis, thus resulting in poor treatment effects and low‐survival rates 2. Various factors have been involved in the aggressive process of PCa, and especially those related to the key signalling transduction or regulated by non‐coding RNAs (ncRNAs) contribute to PCa progression 3 Therefore, it is essential to gain insight into understanding the molecular mechanisms of how ncRNAs modulate PCa and identify novel therapeutic targets for the treatment of PCa.

MicroRNAs (miRNAs) are a class of small ncRNAs consisting of about 18–25 nucleotides and regulate gene expression in many physiological and pathological conditions 4. They can function as oncogenes or tumour suppressors involved in multi‐step tumorigenesis 5, of which miR‐15b exerts a dual function in different types of cancer. Some studies show that miR‐15b expression is down‐regulated in tongue cancer 6, glioma 7, glioblastoma 8, ovarian cancer 9, osteosarcoma 10 and hepatocellular carcinoma (HCC) 11, 12, and its low expression is associated with the poor survival 7, 8, 9, 10 and recurrence 11 of patients with cancer. In addition, the serum level of miR‐15b is decreased in non‐small cell lung cancer (NSCLC) 13 and HCC 14, indicating a potential marker for diagnosis of them. Functionally, miR‐15b inhibits cell proliferation, invasion, metastasis and cycle progression 6, 8, 11, induces cell apoptosis 11, 15 and reverses multidrug resistance 6, 10 by targeting multiple genes such as Wee1, IGF1R and Rab1A 8, 10, 15, while miR‐15b deletion promotes B‐cell malignancies 16, suggesting that miR‐15b may function as a tumour suppressor in cancer.

However, other studies reveal that miR‐15b expression is up‐regulated in lung adenocarcinoma 17, glioma 18, mantle cell lymphoma 19 and uterine leiomyoma 20 and associates with poor prognosis and malignant progression of patients with glioma 17 and melanoma 21. Ectopic expression of miR‐15b is linked to chromosomal changes leading to cervical carcinogenesis 22, enhances cell proliferation 21, cisplatin resistance 16, epithelial‐mesenchymal transition (EMT) and metastasis in pancreatic cancer 23 and predicts the brain metastases from melanoma 24. These studies indicate that miR‐15b acts as an oncogene in cancer.

Though miR‐15b expression has been reported in blood from patients with PCa 25, the molecular mechanisms by which miR‐15b contributes to PCa remain unclear. In this study, we analysed the correlation of miR‐15b expression with clinicopathological characteristics and prognosis of patients with PCa and clarified the function and found that miR‐15b promoted the tumorigenesis of PCa by targeting RECK.

## Materials and Methods

### Materials

PCa cell lines (PC3 and 22RV1) used in our experiment were from Laboratory of Urology, Huadong Hospital. The materials and reagents used in this study were summarized in Table S1.

### Clinical data

The clinical data for 495 cases of patients with PCa and 52 adjacent normal tissues as well as the relative expression levels of miR‐15b and RECK were downloaded from The Cancer Genome Atlas 2015 RNA sequencing database (https://genome-cancer.ucsc.edu). Among the 495 patients with PCa, 387 cases had the intact clinicopathological and prognostic information for further analysing the association of miR‐15b with overall survival and recurrence of patients with PCa.

### Cell culture and transfection

PCa cells were cultured with DMEM medium mixed with 10% FBS and 1% penicillin (100 U/ml) and streptomycin (100 μg/ml) in a humidified atmosphere containing 5% CO_2_ at 37°C. Plasmid‐mediated miR‐15b or RECK overexpression vectors and lentivirus‐mediated miR‐15b shRNA vector as well as cell transfection were conducted as previously described 15.

### Quantitative real‐time PCR (qRT‐PCR)

qRT‐PCR analysis was used to assess the expression levels of miR‐15b and RECK in PCa cells. The primers of miR‐15b, U6, RECK and GAPDH were listed in Table S2 A detailed description of the manipulation steps of qRT‐PCR analysis was indicated in Appendix S1

Western blot analysis, cell viability assay, colony formation assay, Transwell assay, dual‐luciferase reporter assay, animal experiments and statistical analysis were shown in Appendix S1

## Results

### The expression level of miR‐15b was increased in PCa samples

**Figure 1:**
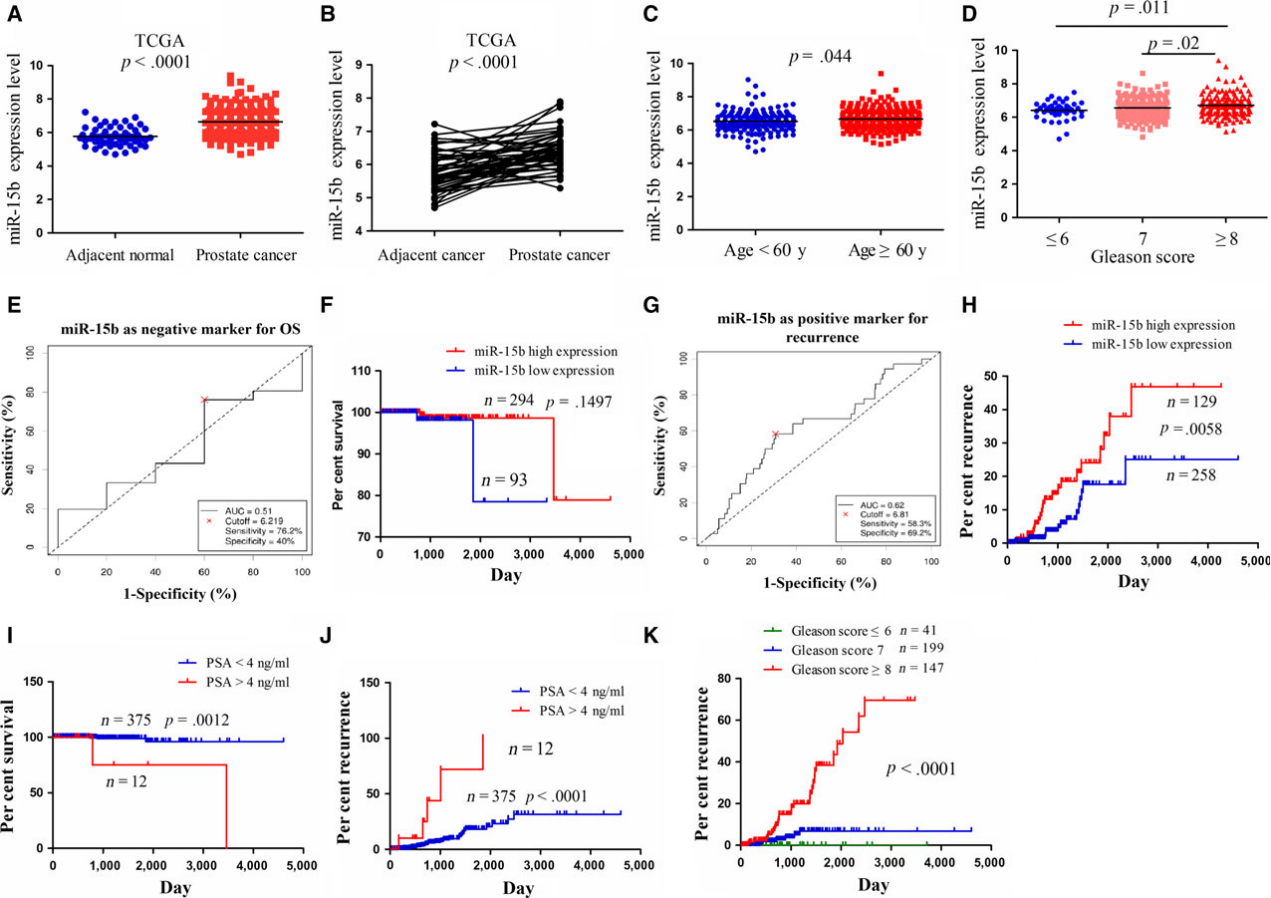
The expression of miR‐15b was up‐regulated in PCa tissues and was associated with tumour recurrence. (**A**) The expression of miR‐15b in 495 PCa patient samples and 52 normal tissues indicated by TCGA data set. (**B**) The expression of miR‐15b in paired PCa tissues (*n* = 52). (**C, D**) The expression levels of miR‐15b in PCa patients with different age and Gleason scores. (**E, G**) The cut‐off values of miR‐15b were determined by the patients’ overall survival time or disease‐free survival time, survival status and miR‐15b expression level. (**F, H**) The correlation of miR‐15b high expression or low expression with overall survival rate and recurrence rate of patients with PCa. (**I, J**) The correlation of PSA serum level with overall survival rate and recurrence rate of patients with PCa. (**K**) The correlation of Gleason scores with the tumour recurrence rate of patients with PCa.

To estimate the expression level of miR‐15b in human PCa tissues, TCGA cohort was used to detect it in PCa and adjacent normal tissues, indicating that miR‐15b expression was markedly up‐regulated in PCa tissues (*n* = 495) compared to the adjacent normal tissues (*n* = 52) (*P* < 0.0001, Fig. 1A) as well as in paired PCa tissues (*n* = 52) (*P* < 0.0001, Fig. 1B). To unveil whether miR‐15b has the differential expression in patients with PCa of different ages and Gleason scores, we evaluated its expression level in PCa tissues from the patients with age ≥60 years (*n* = 225) or age <60 years (*n* = 162) and Gleason score ≤6 (*n* = 41), =7 (*n* = 199) and ≥8 (*n* = 147), indicating that miR‐15b expression was positively correlated with the age and Gleason score of patients with PCa (each *P* < 0.05; Fig. 1C, D).

### High expression of miR‐15b was associated with Gleason score and tumour recurrence in patients with PCa

We analysed the relationship between miR‐15b expression and clinicopathologic parameters and prognosis of patients with PCa. As shown in Table 1, it was illustrated that miR‐15b expression was positively correlated with age (*P* = 0.049) and Gleason score (*P* = 0.03) but had no correlation with lymphatic metastasis and pre‐operative PSA and pathological T stage in patients with PCa (each *P* > 0.05).

**Table 1:** The correlation of miR‐15b expression with clinicopathologic characteristics of patients with prostate cancer

Variables	Cases (*n*)	NEAT1		*P* value
		High	Low	
Total	387	129	258	
Age (years)				
≥60	225	84	141	0.049
<60	162	45	117	
Gleason score				
≤6	41	8	33	0.030
7	199	62	137	
≥8	147	59	88	
Lymphatic metastasis				
Positive	326	114	212	0.115
Negative	61	15	46	
Pre‐operative PSA (ng/ml)				
<4	12	6	6	0.214
>4	375	123	252	
Pathological T stage				
pT2	165	47	118	0.124
pT3	216	81	135	
pT4	6	1	5	

Then, we applied the cut‐off finder (http://molpath.charite.de/cutoff/load.jsp) to define the cut‐off value of miR‐15b in patients with PCa (Fig. 1E). According to the recurrence time, recurrence status and miR‐15b expression level, the cut‐off value of miR‐15b was determined, and the patients were classified into two groups: miR‐15b high expression (cut‐off ≥ 6.81) or low expression group (cut‐off < 6.81; Fig. 1G). Furthermore, Kaplan–Meier (KM) survival analysis displayed no correlation of miR‐15b expression with the survival of patients with PCa (*P* > 0.05, Fig. 1F), while KM recurrence curve showed that the patients with miR‐15b high expression had higher recurrence rate than those with low expression (*P* = 0.0058, Fig. 1H). Multivariate analysis indicated that miR‐15b expression could not act as an independent prognostic factor for overall survival (Table S3) and recurrence (Table S4) of patients with PCa, but pre‐operative PSA or Gleason score was associated with overall survival (Fig. 1I) and recurrence (Fig. 1J, K), and acted as independent factors for them in patients with PCa (Table S3, S4).

### The effects of miR‐15b on PCa cell growth and invasion

**Figure 2:**
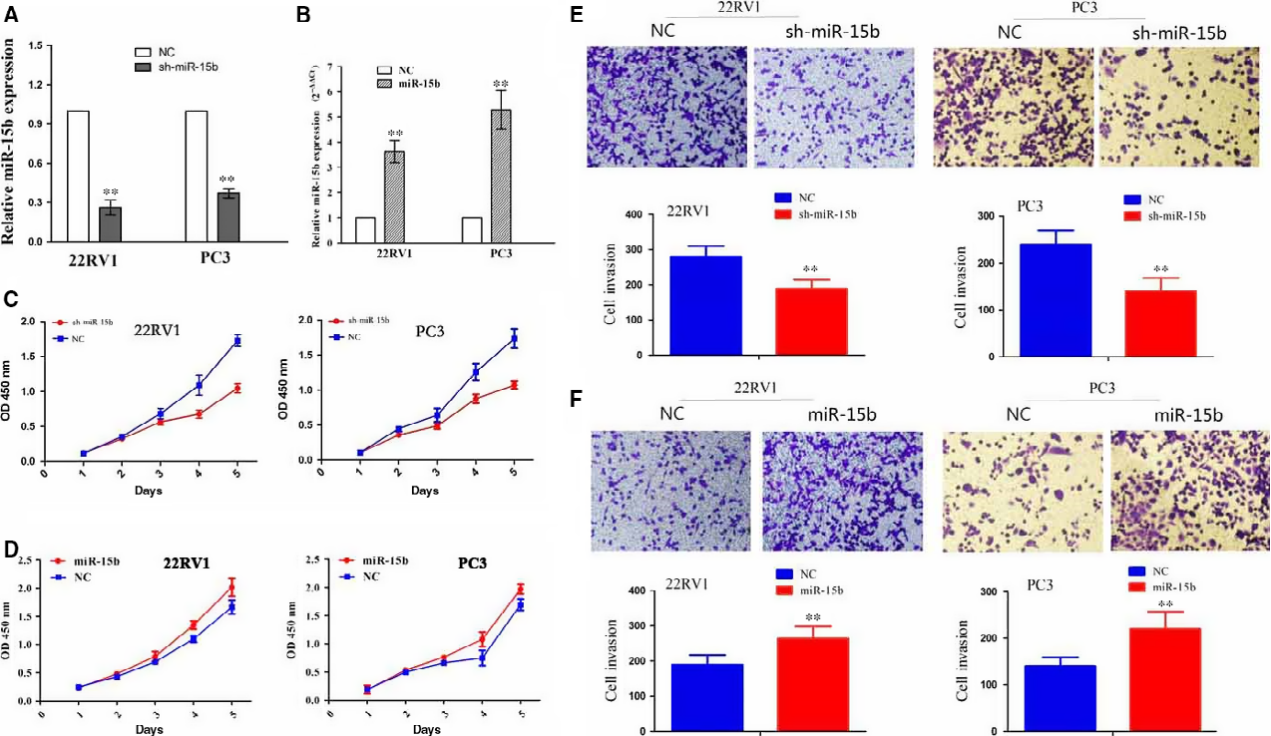
Effects of miR‐15b on PCa cell proliferation and invasion. (**A, B**) The transfection efficiency of sh‐miR‐15b or miR‐15b overexpression in 22RV1 and PC3 cells indicated by qPCR. (**C, D**) The effects of miR‐15b knockdown or overexpression on cell proliferation indicated by MTT assay. (**E, F**) The effects of miR‐15b knockdown or overexpression on cell invasive potential. **P < 0.01, each group *n* = 3, Error bars, mean ± S.E.M.

To further clarify the function of miR‐15b in PCa cells, we transfected miR‐15b shRNA lentivirus or overexpression plasmid intro 22RV1 and PC3 cell lines. After transfection for 48 hrs, miR‐15b expression levels were examined by qRT‐PCR, which indicated a decreased expression in sh‐miR‐15b group (Fig. 2A) but an increased expression in miR‐15b group (Fig. 2B) compared with the negative control group (**P < 0.01). Then, MTT and Transwell assays showed that knockdown of miR‐15b markedly suppressed cell proliferation activity (Fig. 2C) and invasive potential (Fig. 2E), but overexpression of miR‐15b promoted cell proliferation (Fig. 2D) and invasive potential (Fig. 2F) in PCa cells (**P < 0.01).

### RECK was identified as a target of miR‐15b in PCa cells

**Figure 3:**
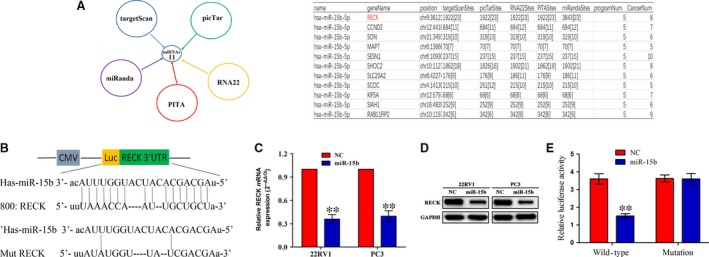
RECK gene was identified as a direct target of miR‐15b in PCa cells. (A) Eleven target genes of miR‐15b were identified by five kinds of miRNA forecasting tools in more than five kinds of cancer tissues, of which RECK was considered as a most suitable candidate target gene due to its highest binding capacity. (**B**) Diagrams demonstrated the miR‐15b putative binding sites and corresponding mutant sites of RECK. (**C, D**) The expression levels of RECK were examined after transfection with miR‐15b by qPCR and Western blotting assays. (**E**) Luciferase activity of RECK (wide type or mutation) was evaluated after miR‐15b transfection for 24 hrs. **P < 0.01, each group *n* = 3, Error bars, mean ± S.E.M.

To nail down the regulation mechanism of miR‐15b in PCa cells, we identified the potential target genes of miR‐15b in cancer tissues using the publicly available bioinformatic analysis. As a result, 11 target genes were discovered to have the potential to bind to miR‐15b, as predicted by targetScan, PicTar, RNA22, PITA and miRanda (Fig. 3A). Among these genes, reversion‐inducing cysteine‐rich protein with Kazal motifs (RECK) was considered to have the greatest potential to bind to miR‐15b. To testify whether miR‐15b can directly bind to the 3′ UTR of RECK gene, we cloned the wide type 3′ UTR or the mutant 3′ UTR sequence of RECK gene (Fig. 3B) into the luciferase reporter vector for transfection into 22RV1 and PC3 cells. The luciferase gene report assay was used to detect the luciferase activity of 3′ UTR of RECK, and qRT‐PCR and Western blot assays were conducted to evaluate the effects of miR‐15b on the expression of RECK. Our results showed that miR‐15b overexpression not only decreased the expression of RECK in PCa cells at the mRNA (Fig. 3C) and protein levels (Fig. 3D), but also reduced the luciferase activity of wide type 3` UTR of RECK but had no effect on that of mutation 3` UTR of RECK (Fig. 3E).

### RECK repressed cell proliferation and invasion of PCa cells

**Figure 4:**
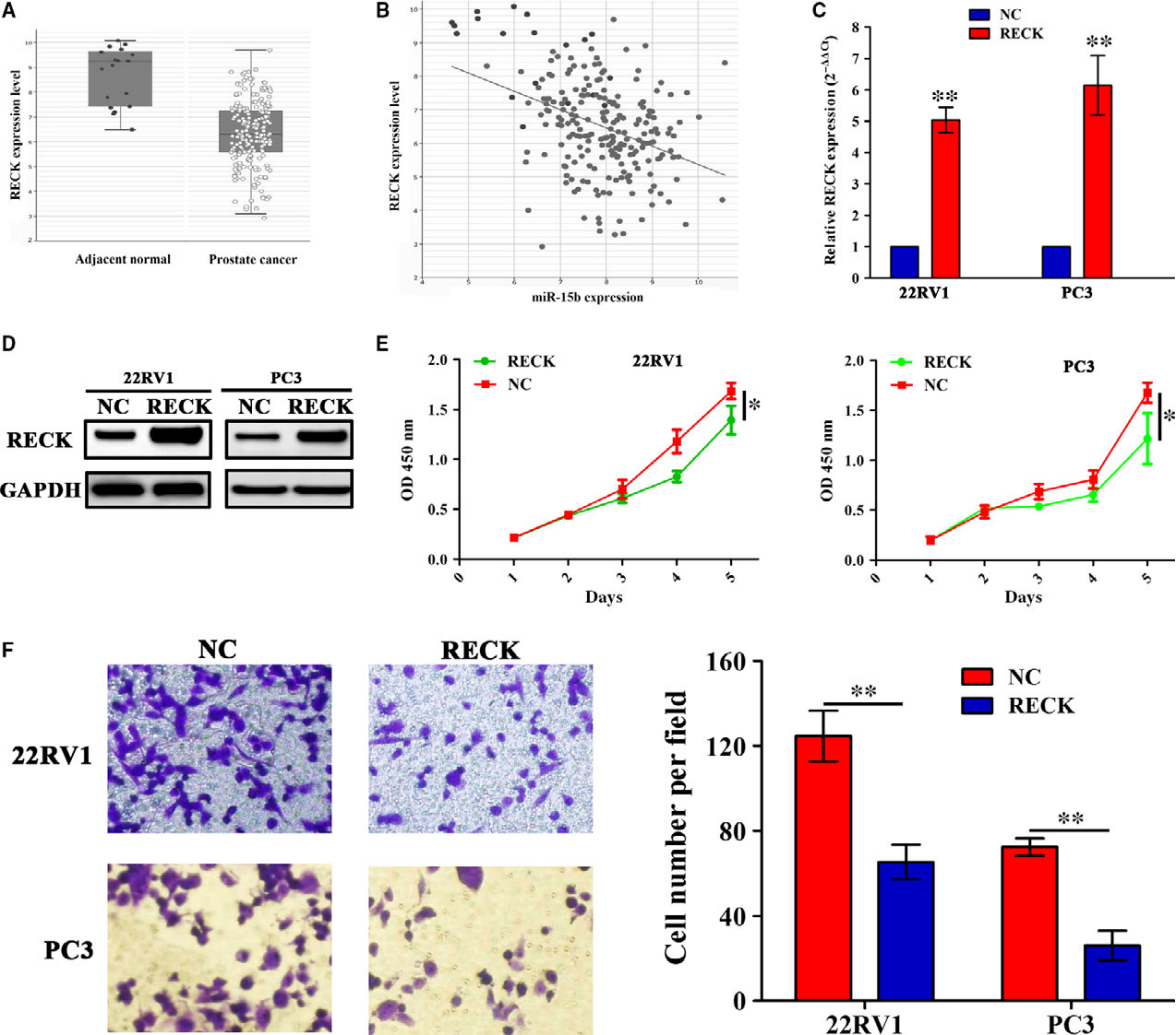
Enforced expression of RECK inhibited PCa cell proliferation and invasion. (**A**) The expression of RECK in PCa tissues (*n* = 208) and the adjacent normal tissues (*n* = 19) shown by TCGA data (*P* < 0.0001). (**B**) The correlation of miR‐15b expression with RECK in patients with PCa (*n* = 208; *r* = −0.38, *P* < 0.0001). (**C, D**) The transfection efficiency of RECK overexpression was determined by qPCR and Western blot assays. (**E, F**) The effects of RECK overexpression on cell proliferation and invasive potential indicated by MTT and Transwell assays. *P < 0.05, **P < 0.01, each group *n* = 3, Error bars, mean ± S.E.M.

To clarify the correlation of miR‐15b with RECK expression in PCa tissues, we first evaluated the expression level of RECK in PCa tissues in TCGA cohort, which indicated that RECK expression level was significantly decreased in PCa tissues compared with the adjacent normal tissues (*P* < 0.0001, Fig. 4A). The Spearman correlation analysis revealed the negative correlation of miR‐15b with RECK expression in PCa tissues (*r* = −0.38, *P* < 0.0001) (Fig. 4B). Then, we transfected RECK overexpression plasmid into 22RV1 and PC3 cells and defined its transfection efficiency by qRT‐PCR (Fig. 4C) and Western blotting analysis (Fig. 4D). Enforced expression of RECK significantly inhibited cell proliferation (Fig. 4E) and invasive potential (Fig. 4F) in PCa cells.

### RECK overexpression counteracted the tumour growth caused by miR‐15b in PCa cells

**Figure 5:**
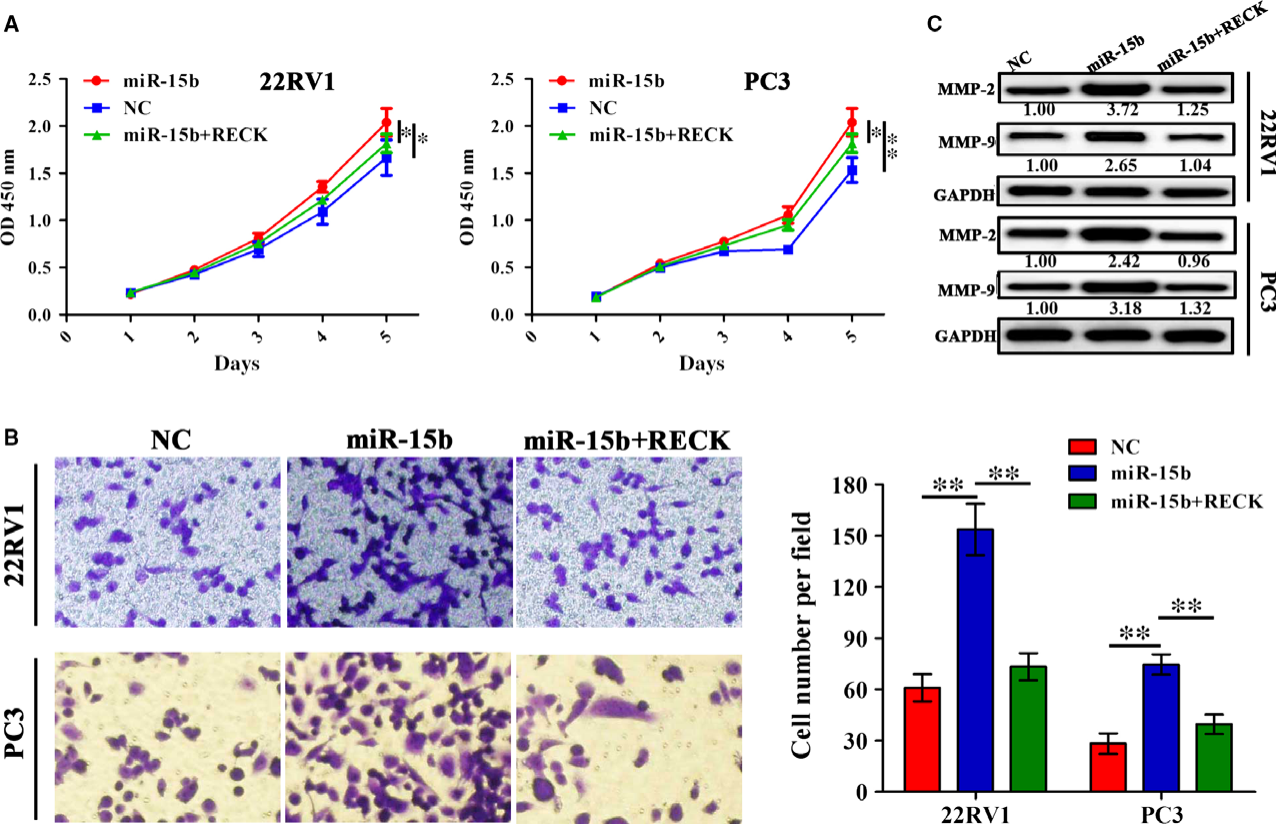
RECK overexpression rescued the tumour‐promoting effects by miR‐15b in PCa cells. (**A, B**) The effects of RECK overexpression on cell proliferation and invasion in miR‐15b‐transfected 22RV1 and PC3 cells indicated by MTT and Transwell assays. (**C**) The effects of RECK overexpression on the protein expressions of MMP‐2 and MMP‐9 in miR‐15b‐transfected PCa cells indicated by Western blot analysis. **P < 0.01, each group *n* = 3, Error bars, mean ± S.E.M.

To investigate whether RECK overexpression can counteract the tumour growth caused by miR‐15b, we cotransfected RECK and miR‐15b overexpression vectors into 22RV1 and PC3 cells and found that RECK overexpression attenuated cell proliferation (Fig. 5A) and invasion promoted by miR‐15b (Fig. 5B). Moreover, the expression levels of MMP‐2/‐9, downstream regulators of RECK, were evaluated by Western blotting, implying that miR‐15b increased the expression levels of MMP‐2/‐9, but RECK overexpression reversed this effect in 22RV1 and PC3 cells (Fig. 5C).

### Knockdown of miR‐15b suppressed PC3 xenograft tumour growth

**Figure 6:**
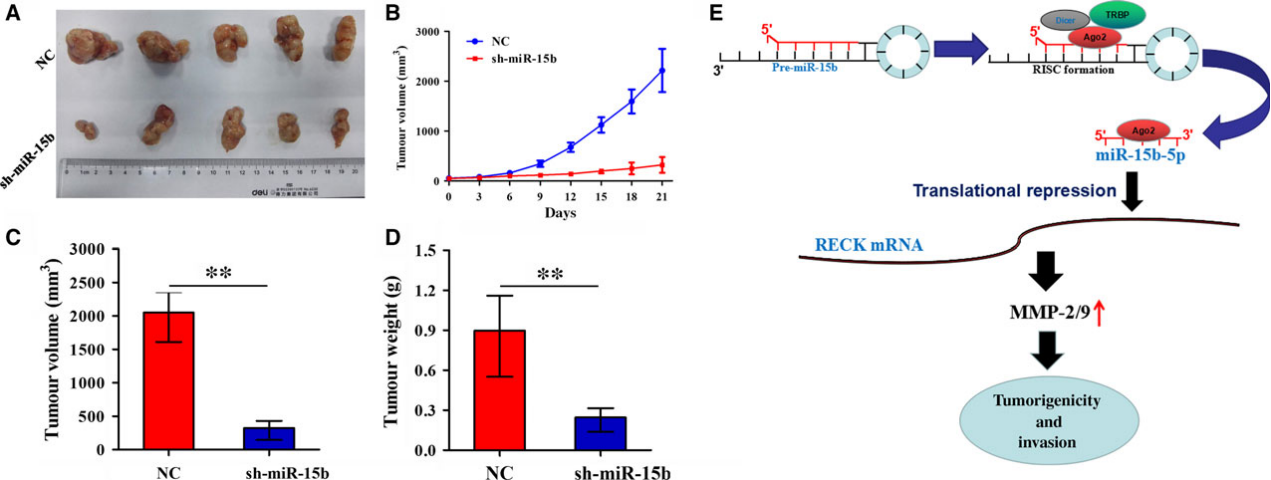
Knockdown of miR‐15b inhibited PC3 xenograft tumour growth. (**A, B**) The effects of miR‐15b knockdown on tumour proliferation activity of PC3 xenograft tumours. (**C, D**) The effects of miR‐15b knockdown on the average volumes and weight in xenograft tumours. **P < 0.01, each group *n* = 5, Error bars, mean ± S.E.M. (**E**) miR‐15b decreased the transcriptional level of RECK gene by binding to its 3′ UTR, and up‐regulated the expression of MMP‐2/‐9, thereby contributing to the tumorigenesis and invasion of PCa.

Having verified the tumour‐promoting effects of miR‐15b *in vitro,* we further observed its effects on cell growth *in vivo*. A subcutaneous PC3 xenograft tumour model was constructed to investigate the tumour growth ability influenced by miR‐15b, which showed that the proliferative activities of PC3 xenograft tumours were markedly lowered by silencing of miR‐15b expression (Fig. 6A, B). Further, the average weight and volumes of the tumours in sh‐miR‐15b group were remarkably decreased compared with the NC group (Fig. 6C, D).

## Discussion

Aberrant regulation of miR‐15b plays a key role in cell proliferation, angiogenesis and metastasis in a variety of tumours 26. According to the previous studies, miR‐15b has displayed decreased expression 6, 7, 8, 9, 10, 11, 12 or increased expression 17, 18, 19, 20, 21 in human tumours. Even, miR‐15b harbours the opposite expression levels in the same tumour tissues, such as glioma 7, 18, indicating that its expression level is related with the specific types of tumour tissues. Current studies show that miR‐15b is highly expressed in the blood of PCa 25, but its association with clinicopathological characteristics and prognosis of PCa patients remains unknown. In this study, we found that miR‐15b expression level was significantly increased in PCa tissues and was positively associated with the Gleason score and tumour recurrence, indicating a poor prognosis in PCa, which corroborated the previous studies in glioma 17 and melanoma 21. These studies suggest that miR‐15b may act as a potential biomarker for PCa.

Functionally, it has been shown that miR‐15b may have a dual role in cancer, acting as a tumour suppressor 6, 8, 11, 15, 16 or an oncogene 16, 21, 23. But, the function of miR‐15b in human PCa is still elusive. In our study, it was found that knockdown of miR‐15b suppressed cell proliferation and invasion *in vitro* and *in vivo*, but enforced expression of miR‐15b promoted cell proliferation and invasion in PCa cells, suggesting that miR‐15b might function as an oncogene in PCa cells.

The reversion‐inducing cysteine‐rich protein with Kazal motifs (RECK) is considered as a tumour suppressor associated with low invasiveness and favourable prognosis in cancers 27, 28, 29, 30 and suppresses tumour invasive and metastatic potential 31, 32. Moreover, multiple miRNAs including miR‐21, miR‐15a, miR‐200b/c, miR‐96 and miR‐221 have been proved to promote tumour growth and metastasis *via* targeting RECK 33, 34, 35, 36, 37. Intriguingly, we also identified RECK as a direct target of miR‐15b in PCa cells and verified that it was negatively associated with miR‐15b expression in PCa tissues. Overexpression of RECK reduced cell growth and invasion and abrogated the tumour‐promoting effect and MMP‐2/‐9 expression caused by miR‐15b, which was consistent with previous studies in cancer cells 20, 33, 38 indicating that miR‐15a might promote the tumorigenicity of PCa cells *via* targeting RECK (Fig. 6E).

In short, our results demonstrate that miR‐15b expression is markedly up‐regulated in PCa tissues and correlates with Gleason score and recurrence of PCa patients. Enforced expression of miR‐15b promotes the tumorigenicity and invasion of PCa cells through targeting RECK and may represent a potential marker for patients with PCa.

## Conflicts of Interest

The authors declare that they have no competing interests.

## Supporting information


**Table S1** List of Materials and Regents.
**Table S2** List of primers.
**Table S3** Cox regression analysis of miR‐15b expression as overall survival predictor for patients with prostate cancer.
**Table S4** Cox regression analysis of miR‐15b expression as recurrence predictor for patients with prostate cancer.

**Appendix S1** Materials and Methods.
