# Supplementary material for: miR‐15b‐5p facilitates the tumorigenicity by targeting RECK and predicts tumour recurrence in prostate cancer
Source: J Cell Mol Med. 2018 Jan 24;22(3):1855–63. doi: 10.1111/jcmm.13469 (PMC5824417; doi:10.1111/jcmm.13469)
Supplement: Supplementary file 1 — Table S1 List of Materials and Regents. Table S2 List of primers. Table S3 Cox regression analysis of miR‐15b expression as overall survival predictor for patients with prostate cancer. Table S4 Cox regression analysis of miR‐15b expression as recurrence predictor for patients with prostate cancer. [file JCMM-22-1855-s001.docx]

Table S1 List of Materials and Regents

| Materials and reagents | Company Name | Origin and Country |
| --- | --- | --- |
| miR-15b overexpression plasmid | Genechem | Shanghai, PR, China |
| miR-15b shRNA lentivirus | Genechem | Shanghai, PR, China |
| RECK overexpression plasmid | Genechem | Shanghai, PR, China |
| negative control | Genechem | Shanghai, PR, China |
| virion-packaging elements | Genechem | Shanghai, PR, China |
| All antibodies | Cell Signaling Technologies | Beverly, MA, USA |
| Dulbecco’s Modified Eagle medium (DMEM) | Thermo Fisher Scientific Inc | Waltham, MA, USA |
| fetal bovine serum (FBS) | Thermo Fisher Scientific Inc | Waltham, MA, USA |
| 3-(4,5)-dimethylthiahiazo (-z-yl)-3,5-di- phenytetrazoliumromide (MTT) | Dingguo biology | Shanghai, PR, China |
| TRIzol Reagent | Invitrogen | Carlsbad, CA, USA |
| Lipofectamine 2000 | Invitrogen | Carlsbad, CA, USA |
| M-MLV Reverse Transcriptase | Promega | Madison, WI, USA |
| SYBR Green Master Mixture | Takara | Otsu, Japan |
| RNase A | KeyGEN biology | Nanjing, PR, China |
| ECL-PLUS/Kit | GE Healthcare | Piscataway, NJ, USA |
| miRNeasy mini kit | invitrogen | Carlsbad, CA, USA |

Table S2 List of primers

| Gene | Sense primer | Antisense primer |
| --- | --- | --- |
| miR-15b | 5′-ATGAACTTTCTCTGTCTTGG-3′ | 5′-TCACCG CCTCGGCTTGTCACA-3′ |
| U6 | 5′-CTCGCTTCGGCAGCACA-3′ | 5′-AACGCTTCACGAATTTGCGT-3′ |
| RECK | 5′-TGCAAGCAGGCATCTTCAAA-3′ | 5′-ACCGAGCCCATTTCATTTCTG-3′ |
| GAPDH | 5′-GCACCGTCAAGGCTGAGAAC-3′ | 5′-TGGTGAAGACGCCAGTGGA-3′ |

Table S3 Cox regression analysis of miR-15b expression as overall survival

predictor for patients with prostate cancer

| Variables | Univariate Cox regression analysis | |  | Multivariate Cox regression analysis | |
| --- | --- | --- | --- | --- | --- |
|  | RR (95% CI) | *P* value |  | RR (95% CI) | *P* value |
| *Age (years)* |  |  |  |  |  |
| <60 vs. ≥60 | 1.280 (0.212 to 7.725) | 0.7884 |  | NA | NA |
| *Gleason score* |  |  |  |  |  |
| ≤6 vs. 7 vs ≥8 | 6.224 (0.755 to 51.317) | 0.089 |  | NA | NA |
| *Lymphatic metastasis* |  |  |  |  |  |
| Positive vs. Negative | 24.912 (0.321 to 124.232) | 0.554 |  | NA | NA |
| *Preoperative PSA (ng/ml)* |  |  |  |  |  |
| <4 vs. >4 | 16.483 (2.326 to 116.824) | 0.005 |  | 16.483 (2.326 to 116.824) | 0.005 |
| *Pathological T stage* |  |  |  |  |  |
| pT2vs. pT3 vs. pT4 | 2.575 (1.182 to 4.257) | 0.301 |  | NA | NA |
| *miR-15b expression* |  |  |  |  |  |
| High VS. Low | 0.693 (0.137 to 3.506) | 0.658 |  | NA | NA |

Table S4 Cox regression analysis of miR-15b expression as recurrence

predictor for patients with prostate cancer

| Variables | Univariate Cox regression analysis | |  | Multivariate Cox regression analysis | |
| --- | --- | --- | --- | --- | --- |
|  | RR (95% CI) | *P* value |  | RR (95% CI) | *P* value |
| *Age (years)* |  |  |  |  |  |
| <60 vs. ≥60 | 1.046 (0.540 to 2.025) | 0.894 |  | NA | NA |
| *Gleason score* |  |  |  |  |  |
| ≤6 vs. 7 vs ≥8 | 6.578 (2.961 to 14.213) | <0.001 |  | 5.367 (2.293 to 12.564) | <0.001 |
| *Lymphatic metastasis* |  |  |  |  |  |
| Positive vs. Negative | 1.721 (0.527 to 5.621) | 0.369 |  | NA | NA |
| *Preoperative PSA (ng/ml)* |  |  |  |  |  |
| <4 vs. >4 | 9.721 (3.712 to 25.457) | <0.001 |  | 4.387 (1.634 to 11.775) | 0.003 |
| *Pathological T stage* |  |  |  |  |  |
| pT2vs. pT3 vs. pT4 | 2.243 (1.182 to 4.257) | 0.013 |  | 1.166 (0.535 to 2.542) | 0.699 |
| *miR-15b expression* |  |  |  |  |  |
| High VS. Low | 1.591 (0.956 to 2.648) | 0.074 |  | 1.161 (0.682 to 1.976) | 0.583 |
