# Supplementary material for: miR‐15b‐5p facilitates the tumorigenicity by targeting RECK and predicts tumour recurrence in prostate cancer
Source: J Cell Mol Med. 2018 Jan 24;22(3):1855–63. doi: 10.1111/jcmm.13469 (PMC5824417; doi:10.1111/jcmm.13469)
Supplement: Supplementary file 2 — Appendix S1 Materials and Methods. [file JCMM-22-1855-s002.docx]

**Quantitative Real-time PCR (qRT-PCR)**

Total RNA exaction, reverse transcription and cDNA amplification was manipulated depending on the protocols provided by the manufacturers, of which miR-15b exaction was based on the protocols provided by miRNeasy mini kit. The primers of miR-15b, U6, RECK and GAPDH were listed in Table S2. Data were analysed using the comparative Ct method (2-△△Ct). Three separate experiments were performed for each clone.

**Western blot analysis**

PCa cells were harvested and extracted using lysis buffer (Tris-HCl, SDS, Mercaptoethanol, Glycerol)., and the experimental protocols for western blot analysis was performed as previously reported [26].

MTT

**Cell viability assay**

PCa cells transfected with miR-15b, miR-15b shRNA and RECK were incubated in 96-well-plates at a density of 2×10^3^ cells. Cells were pretreated with 10μl of MTT dye each day. After incubation for 4 h, 100μl of DMSO were added into the cells for 15 min. The color reaction was measured at 570 nm using an Enzyme Immunoassay Analyzer (Bio-Rad, Hercules, CA).

**Colony formation assay**

PCa cells transfected with miR-15b, miR-15b shRNA and RECK were trypsinized and reseeded into 6-well plate. The number of cell colonies in control and treatment groups were counted after a week and were stained with crystal violet.

**Transwell invasion assay**

Transwell filters were coated with matrigel (3.9 μg/μl, 60-80 μl) on the upper surface of a polycarbonic membrane (diameter 6.5 mm, pore size 8 μm). After incubating at 37°C for 30 min, the matrigel solidified and served as the extracellular matrix for analysis of tumor cell invasion. Harvested cells (1×10^5^) in 100 μl of serum free DMEM were added into the upper compartment of the chamber. A total of 200 μl conditioned medium derived from NIH3T3 cells was used as a source of chemoattractant, and was placed in the bottom compartment of the chamber. After 24 h incubation at 37°C with 5% CO_2_, the medium was removed from the upper chamber. The non-invaded cells on the upper side of the chamber were scraped off with a cotton swab. The cells that had migrated from the matrigel into the pores of the inserted filter were fixed with 100% methanol, stained with Hematoxylin, and mounted and dried at 80°C for 30 min. The number of cells invading through the matrigel was counted in three randomly selected visual fields from the central and peripheral portion of the filter using an inverted microscope (200×magnification). Each assay was repeated three times.

**Dual-luciferase reporter assay**

PCa cells were cultured in 24-well plates. Gene report vector containing wild type 3′-UTR or mutated 3′-UTR of RECK target gene was co-transfected with negative control or miR-15b into the PCa cells. The detained manufacturer’s instructions were referred to previously published [26].

**Animal experiments**

Six-week-old female immune-deficient nude mice were fed in Animal Institute of Chinese Academy of Sciences based on the regulations and internal biosafety and bioethics guidelines of Hua Dong Hospital, Fu Dan University. Mice were injected subcutaneously with 1×10^6^ PC3 cells stably transfected with sh-miR-15b or control cells. The length and width of the Xenogratft tumors was measured with a caliper every other day.

**Statistical analysis**

SPSS 22.0 was applied for the statistical analysis of the values, which were recorded as the mean ±SEM. Paired or independent t test was used to analyse the differential change in each group. Statistical significance was set at *P* < 0.05.
